# Is there a role for music in the ICU?

**DOI:** 10.1186/s13054-014-0663-1

**Published:** 2015-01-16

**Authors:** Jeffrey D DellaVolpe, David T Huang

**Affiliations:** Department of Critical Care Medicine, University of Pittsburgh, 642A Scaife Hall, 3550 Terrace Street, Pittsburgh, PA 15261 USA; Department of Emergency Medicine, University of Pittsburgh, 3550 Terrace Street, Pittsburgh, PA 15261 USA

## Expanded abstract

### Citation

Chlan LL, Weinert CR, Heiderscheit A, Tracy MF, Skaar DJ, Guttormson JL, Savik K: **Effects of patient-directed music intervention on anxiety and sedative exposure in critically ill patients receiving mechanical ventilatory support: a randomized clinical trial.***JAMA* 2013, **309:**2335**–**2344.

### Background

Alternatives to sedative medications, such as music, may alleviate the anxiety associated with ventilatory support.

### Methods

*Objective:* The aim of the study was to test whether listening to self-initiated patient-directed music (PDM) can reduce anxiety and sedative exposure during ventilatory support in critically ill patients.

*Design:* This study was a randomized clinical trial.

*Setting:* In 12 ICUs of five hospitals in the Minneapolis–St Paul, Minnesota area, 373 patients receiving acute mechanical ventilatory support for respiratory failure were enrolled between September 2006 and March 2011. Of the patients included in the study, 86% were white, 52% were female, and the mean age was 59 years. The patients had a mean Acute Physiology, Age and Chronic Health Evaluation III score of 63 and a mean of 5.7 study days.

*Interventions:* The patients received self-initiated PDM (*n* =126) with preferred selections tailored by a music therapist, or self-initiated use of noise-canceling headphones (NCH; *n* = 122), or usual care (*n* = 125).

*Outcomes:* Daily assessments of anxiety (on a 100 mm visual analog scale) and two aggregate measures of sedative exposure (intensity and frequency) were assessed.

### Results

Patients in the PDM group listened to music for a mean of 79.8 (median (range) 12 (0 to 796)) minutes/day. Patients in the NCH group wore the noise-abating headphones for a mean of 34.0 (median (range), 0 (0 to 916)) minutes/day. The mixed-models analysis showed that, at any time point, patients in the PDM group had an anxiety score that was 19.5 points lower (95% confidence interval, −32.2 to −6.8) than patients in the usual care group (*P* = 0.003). By the fifth study day, anxiety was reduced by 36.5% in PDM patients. The treatment × time interaction showed that PDM significantly reduced both measures of sedative exposure. Compared with usual care, the PDM group had reduced sedation intensity by −0.18 (95% confidence interval, −0.36 to −0.004) points/day (*P* = 0.05) and had reduced frequency by −0.21 (95% confidence interval, −0.37 to −0.05) points/day (*P* = 0.01). The PDM group had reduced sedation frequency by −0.18 (95% confidence interval, −0.36 to −0.004) points/day versus the NCH group (*P* = 0.04). By the fifth study day, the PDM patients received two fewer sedative doses (reduction of 38%) and had a reduction of 36% in sedation intensity.

### Conclusions

Among ICU patients receiving acute ventilatory support for respiratory failure, PDM resulted in greater reduction in anxiety compared with usual care, but not compared with NCH. Concurrently, PDM resulted in greater reduction in sedation frequency compared with usual care or NCH, and greater reduction in sedation intensity compared with usual care but not compared with NCH.

## Commentary

Music is an intriguing but relatively understudied intervention with multiple potential benefits for mechanically ventilated, critically ill patients. As ICU and hospital mortality improve, other patient-centered outcomes such as alleviating pain, discomfort, and anxiety become important to address [1] – not only from a patient care perspective but also due to their role in improving long-term effects, such as post-traumatic stress disorder [2]. Often the response to anxiety and stress amongst patients involves sedation, with common side effects such as bradycardia, hypotension, weakness, and delirium [3]. As a result, the 2013 Society of Critical Care Medicine Guidelines for the Management of Pain, Agitation, and Delirium in the ICU recommend nonpharmacologic interventions, such as music, because they are opioid-sparing, low cost, easy to provide, and safe, while acknowledging that few studies have been published on their effectiveness [4].

Despite the perceived benefits of music, there are very few studies validating its use in critically ill patients. Those studies that have examined the effect of music have only done so in the course of a single listening session, either by observing a beneficial effect in heart rate and respiratory rate [5] or in overall anxiety [6]. The long-term effects remain more uncertain, as one study noted that the decrease in blood pressure observed during a music listening session was accompanied by a corresponding rise after cessation of treatment [7]. A final study found no effect of music on serum biomarkers of the stress response between patients listening to music and those resting quietly [8].

No prior studies, however, have prospectively examined the effects of continued music therapy in ICU patients. This study is a three-arm randomized trial examining the effect of patient-directed music (PDM) on anxiety and sedative exposure in mechanically ventilated patients compared with noise-canceling headphones (NCH) and usual care. Sedation exposure was measured both in terms of intensity (weight-adjusted dose given during a 4-hour block) and frequency (the number of 4-hour blocks during which any sedation was administered) [9]. Because anxiety is directly related to amount of sedation, results were modeled using a mixed-effects analysis to predict anxiety, sedation frequency, and intensity while adjusting for covariates of interest. Overall, PDM was associated with lower anxiety scores, sedation frequency, and sedation intensity compared with usual care. There was no significant reduction in anxiety or sedation intensity for PDM compared with NCH.

This study took on the challenging task of measuring in a randomized, prospective manner an aspect of patient care that is both often neglected and difficult to quantify. Additionally, the study quantified the endpoint of anxiety while separating out the possible confounding effect of sedation. However, while this study provides a good framework for examining the role of music in the ICU patients, several issues remain. First, the eligibility criteria eliminated over 96% of patients evaluated for the study, as patients were invited to participate if they were alert, participating in their daily care routines, appropriately following commands, cognitively intact to participate in the consent process, and had adequate or corrected vision and hearing. While including these criteria was probably necessary so that the study arms could be easily compared without confounders such as acute illness and unresponsiveness, the rigidity of the inclusion and exclusion criteria also detracted from the study’s generalizability. Additionally, sedation was not protocolized or standardized, which could potentially decrease the robustness of the main study outcome measure of sedative exposure [10]. Finally, while a decrease in both anxiety and sedation was demonstrated with the use of PDM over usual care, further studies will be needed to delineate the extent of the benefit of PDM, especially as it related to NCHs, because no difference in anxiety scores or sedation intensity was demonstrated between the PDM and NCH groups.

The role that music should play in the care of ICU patients has yet to be defined. For the patient who meets the study’s rigid inclusion criteria, PDM is a compelling option – music is inexpensive with few adverse effects, is easy to administer, and, based on this study, may reduce anxiety and sedation compared with usual care. The findings of this study underscore the importance of addressing behavioral issues in the ICU to improve patient-centered outcomes. Could music play a broader role in the care of critically ill patients? At this point, further study is required – particularly with regards to the effect of music on populations excluded from this study and the long-term cost and feasibility outside a study setting. The cost may be well justified by the overall cost savings, but this will need to be further defined and quantified in further studies.

## Recommendation

Music has the potential to benefit critically ill patients. However, the lack of evidence of its efficacy in a broad population and the need for further validation discourages its widespread use at the current time.

## Abbreviations

NCH: Noise-canceling headphones
PDM: Patient-directed music
